# Supplementary figures and images for: Expression and Possible Role of Nicotinic Acetylcholine Receptor ε Subunit (AChRe) in Mouse Sperm
Source: Biology (Basel). 2021 Jan 11;10(1):46. doi: 10.3390/biology10010046 (PMC7826850; doi:10.3390/biology10010046)

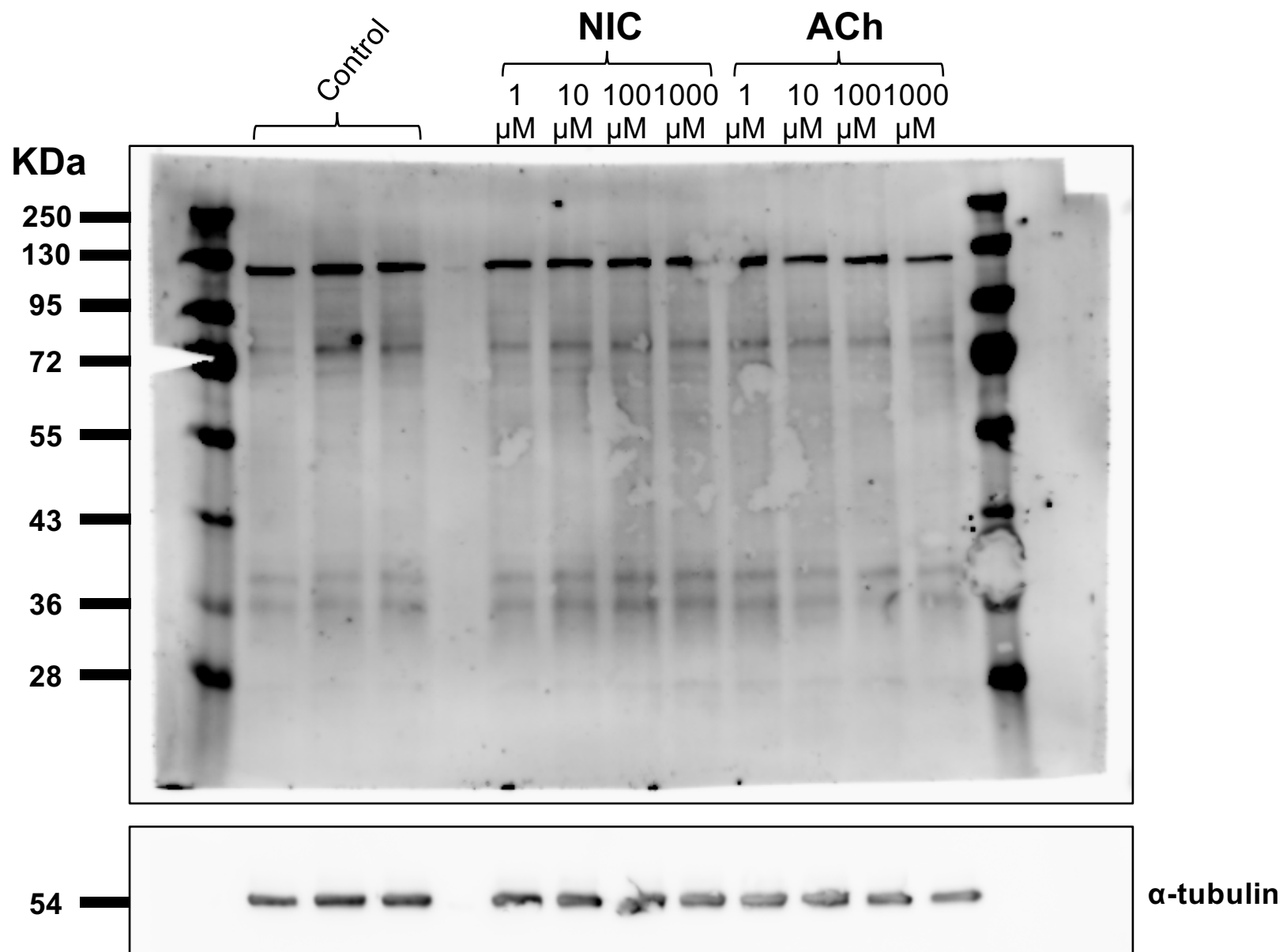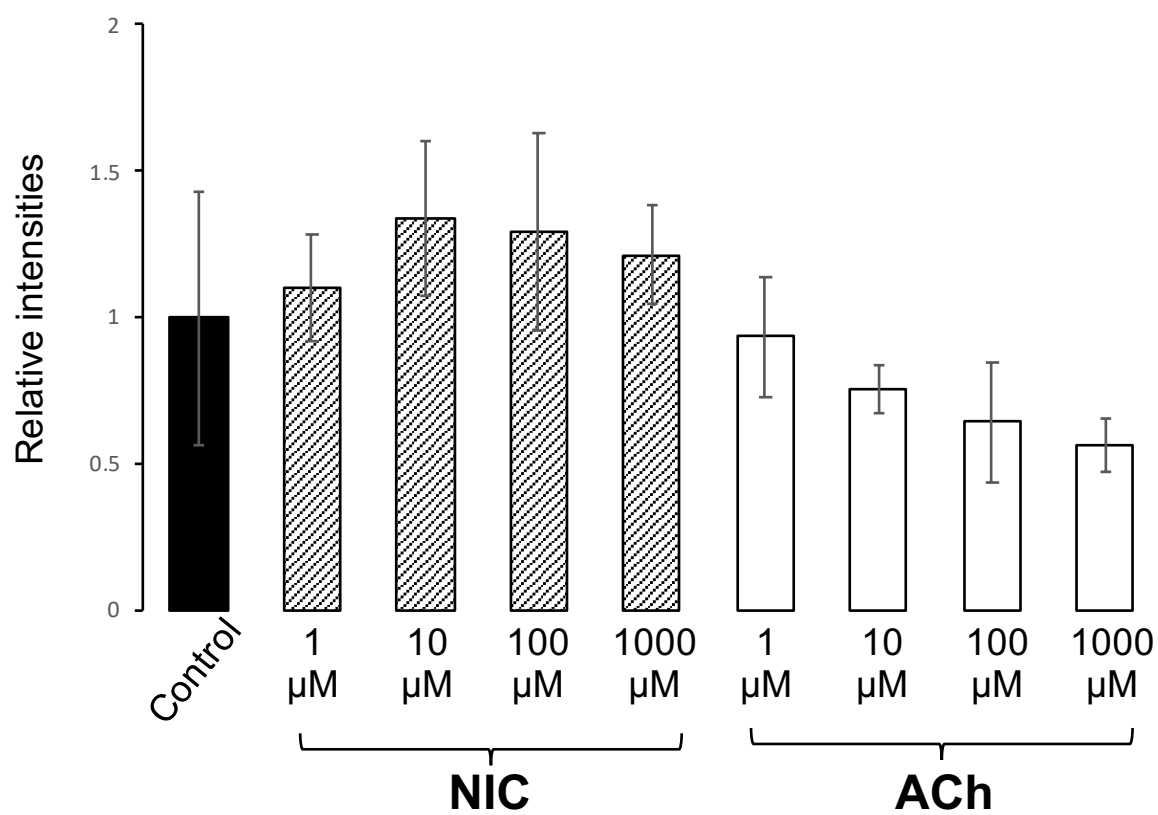

Supplement: Supplementary file 1 [file biology-10-00046-s001.pdf]
